# Knowledge in process? Exploring barriers between epidemiological research and local health policy development

**DOI:** 10.1186/1478-4505-8-26

**Published:** 2010-09-16

**Authors:** Joyce de Goede, Kim Putters, Tom van der Grinten, Hans AM van Oers

**Affiliations:** 1 Academic Collaborative Centre of Public Health Brabant, Tilburg University, Tilburg, the Netherlands; 2 Institute of Health Policy and Management, Erasmus University, Rotterdam, the Netherlands; 3 Department of Public Health Status and Forecasts, National Institute of Public Health and the Environment, Bilthoven, the Netherlands

## Abstract

**Background:**

In the Netherlands municipalities are legally required to draw up a Local Health Policy Memorandum every four years. This policy memorandum should be based on (local) epidemiological research as performed by the Regional Health Services. However, it is largely unknown if and in what way epidemiological research is used during local policy development. As part of a larger study on knowledge utilization at the local level in The Netherlands, an analytical framework on the use of epidemiological research in local health policy development in the Netherlands is presented here.

**Method:**

Based on a literature search and a short inventory on experiences from Regional Health Services, we made a description of existing research utilization models and concepts about research utilization. Subsequently we mapped different barriers in research transmission.

**Results:**

The interaction model is regarded as the main explanatory model. It acknowledges the interactive and incremental nature of policy development, which takes place in a context and includes diversity within the groups of researchers and policymakers. This fits well in the dynamic and complex setting of local Dutch health policy.

For the conceptual framework we propose a network approach, in which we "extend" the interaction model. We not only focus on the one-to-one relation between an individual researcher and policymaker but include interactions between several actors participating in the research and policy process.

In this model interaction between actors in the research and the policy network is expected to improve research utilization. Interaction can obstruct or promote four clusters of barriers between research and policy: expectations, transfer issues, acceptance, and interpretation. These elements of interactions and barriers provide an actual explanation of research utilization. Research utilization itself can be measured on the individual level of actors and on a policy process level.

**Conclusion:**

The developed framework has added value on existing models on research utilization because it emphasizes on the 'logic' of the context of the research and policy networks. The framework will contribute to a better understanding of the impact of epidemiological research in local health policy development, however further operationalisation of the concepts mentioned in the framework remains necessary.

## Background

In the Netherlands in 1989 a new law on collective prevention was approved by parliament: the Public Health Preventive Measures Act (in Dutch abbreviated to WCPV) [1]. This law made the municipalities responsible to protect and promote the health of their population. In 2003 all municipalities became legally required by an amendment of the WCPV to draw up a Local Health Policy Memorandum every four years. To encourage evidence-based policy development, this law required that local health policy should be based upon epidemiological research. Although the WCPV tried to reinforce a renewed collaboration between policy and research, this was not always successful [1,2]. It is largely unknown if and in what way epidemiological research is used during policy development at the local level. Furthermore it is not clear what the reasons are behind (not) using this research.

### Context of Dutch local health policy development

**Figure 1:**
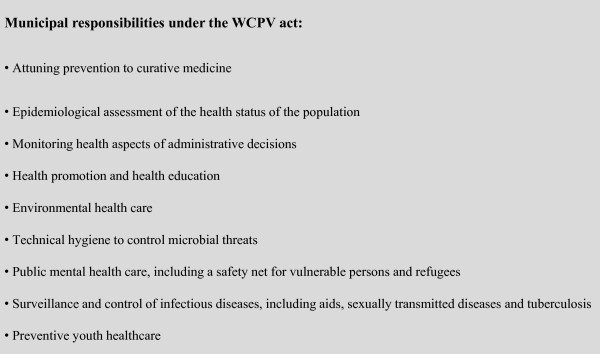
**Textbox 1: Elements of the Public Health Prevention Measures Act**.

Dutch municipalities are responsible for a range of public health tasks, of which "epidemiological assessment of the health status of the population" is one. In figure 1 all WCPV-tasks are presented. Municipalities delegate their public health tasks to a Regional Health Service (RHS).

In total 29 RHSs are active in the Netherlands, covering all Dutch municipalities. The tasks of a RHS are performed by professionals from social medicine, nursing, epidemiology and health promotion. Although the RHS-epidemiologists are assembled in a National Association there is still a large variation in research methods and reporting styles in assessing and reporting the health status of the local population. These differences depend on academic background, personal preferences and organizational structures of the RHS. In past years, most RHS-epidemiologists primarily assess the population health status by describing the public health condition and linking it to preventable risk factors. This population health assessment generally ends with the conclusion that *"something must be done" *[3]. Research concerning *"what should be done" *has less attention in the RHS research setting.

In 2003 an amendment of the WCPV required municipalities to develop and implement a Local Health Policy Memorandum every four years. How this should be done was not pronounced, but three requirements were given: (1) it should be integrated health policy connected with other local policy domains, (2) it should be developed and implemented with actors in the local public health field and (3) it should be based on epidemiological research. As a result of this amendment, the development of a Local Health Policy Memorandum became a complex multi-actor process: decisions in this process had to be made in different settings, by different actors, using different resources [1,2,4-9]. This amendment directed RHS-epidemiologists to deliver *more comparable data *for municipalities and, also to deliver *more usable knowledge *for specific municipalities. Furthermore, a new discipline rose in RHSs: local health policy-advisers who support municipalities with the development of local health policy [2]. Simultaneously on the national level, the ministry of Health, Welfare and Sports drew up a new National Memorandum for prevention [10]. This memorandum was largely based on the public health report from the National Institute of Public Health and the Environment (RIVM), published every four years. These reports and accompanying websites [11] describe the current health status of the Dutch population.

There are three aspects that make the relation between municipalities and their RHS a complex one. First of all, municipalities are the principal funders of the RHS. Dutch RHSs in general serve multiple municipalities, and therefore are directed by more than one. This implies that a RHS performs the same tasks for all municipalities in its region. But these regional tasks have to fit also the specific needs of the individual municipality [2]. The second aspect refers to the range of duties and roles that a municipality expects from the RHS. This can vary from an executive role - carrying out necessary tasks of the WCPV - to an advising role in drafting local health policies. A potential role conflict can appear when, within the RHS, different divisions take different attitudes toward municipalities [2]. The third aspect refers to the communication within and between regional health service and municipalities. There are many (inter)organizational connections, on various management levels. There is an extensive information flow within and between organizations, so a good regulation is necessary in order to avoid misunderstandings.

To summarize the above-mentioned, we can state that the context for the development of local health policy in The Netherlands is a complex one. On the one hand, many actors are involved - and the RHS is one of them - and these actors are also related to and dependent upon each other. On the other hand, national developments influence the local policy processes and outcomes.

### Aim of this study

In recent years growing attention on research utilization in policy processes was seen in Dutch [1,4,6,7,9,12] and international literature [13-15]. However, empirical studies are still scarce and largely outnumbered by theoretically oriented articles. Also in The Netherlands there is hardly any empirical study on the use and impact of epidemiological research on local health policymaking. Therefore an in-depth study on knowledge utilization at the local level in The Netherlands was setup. As part of this study, an analytical framework on the use of epidemiological research in local health policy development in the Netherlands is presented in this article, to be used for further empirical studies in the remainder of the project. To develop the framework, we first provide an overview of explanatory models for research utilization, based on national and international literature. Secondly, we describe barriers between policymakers and researchers, based on national and international literature, and on an inventory of the experiences of Regional Health Service (RHS) epidemiologists in the Netherlands. Thirdly, we discuss the two most appropriate theoretical concepts of research utilization and research impact. Based on these findings we conclude this article with the proposal of an analytical framework for further empirical studies concerning research utilization in local public health policy.

## Methods

### Literature review

We used different search strategies in order to find relevant literature. Firstly we used selected known Dutch studies and dissertations, and international books [1,2,4-6,9,12,13,16,17] on this topic. The Dutch studies and dissertations were mainly used in order to make an analysis of the context of local health policy making. Secondly we searched in different national and international websites http://www.odi.org.uk/RAPID/, http://www.ruru.ac.uk/, http://www.idrc.ca/, http://www.chsrf.ca/home_e.php, http://www.who.int/topics/health_policy/en/, http://www.evipnet.org/php/index.php concerning research utilization and health policy development. Thirdly specific literature was searched using Pubmed and Google Scholar, using the key words "evidenced based policy", "research utilization", "epidemiology" and "local government". Articles and books published between 1975 and 2006 were included in the study. In addition the snowball method was used in order to identify other relevant articles not thrown up by the initial search. After 2006 we followed up the literature by regularly reviewing international websites and relevant international scientific journals (including using RSS feeds). The materials selected for inclusion represent the most relevant dealing with the topics (context of local Dutch health policy, utilization of local epidemiological health research) covered in this article.

### Narratives

To ensure we missed no aspects of research utilization that were not mentioned in literature we conducted an inventory among epidemiologists working in RHSs. By means of the National Association of RHS-epidemiologists, representing 33 RHSs, we asked them by mail to give narratives of (the lack of) research-utilization from their own experience.

We asked them to take a particular case in mind, in which it was irrelevant whether it was an example of "good", "bad" or "non" use of epidemiological research. We asked the epidemiologists about four topics:

• Research context: aim, persons who give the assignment, financiers, collaborative partners, research method;

• Main outcomes of the research, considered important by epidemiologists;

• Follow up given to the results;

• Explanation of this follows up.

We received 25 reactions from 15 RHSs. The narratives were coded by hand based on the overview of barriers found in the literature. We found no barriers, which were not mentioned in literature.

### The construction of the framework

Based on the results from the literature we made a description of existing research utilization models and concepts about research utilization. After this we mapped different barriers in an overview. To make the overview more workable for practitioners from RHSs and policymakers in the field we asked ourselves the question: How far can these barriers be overcome? Therefore we classified them into two groups: (1) barriers at the process level, which can be worked on during the epidemiological research process and are preventable, and (2) barriers at the individual level, which are much harder to tackle because these barriers are hidden and related to personal values and norms of the receivers as well the senders of the research information. From this practical point of view we divided the group of process barriers into the barriers by phase of the research process. Within the group of individual characteristics we distinguished barriers which are negotiable during the policy process and the ones that can only be changed by learning and experience. Subsequently we checked the overview of barriers with the findings of the narratives. We integrated the findings into one framework. In that framework we chose a specific research utilization model, and combined it with the overview of barriers, to make it fit with the specific Dutch policy context. The framework was presented to and discussed with academics and practitioners from our Collaborative Centre Public Health of the University of Tilburg, academics from the Health Governance Group of the Institute of Health Policy and Management of the Erasmus University in Rotterdam, epidemiologists from the National Association of RHSs and policy advisors from the National Association RHSs, all working in public health field in the Netherlands.

## Findings

This section contains three sub-sections. The first sub-section gives a summary of models for explaining research utilization in policymaking found in the literature. The second sub-section gives an overview of possible barriers in research utilization. The third sub-section describes different possibilities to describe research utilization or research impact itself.

### Theoretical explanations for research utilization

Various researchers have created research utilization models or frameworks. In general, these models share the common goal of explaining the apparent gap between research and policy. In general six types of research utilization models can be distinguished. Table 1 shows the main characteristics and shortcomings of each of these models.

**Table 1:** Overview of explanatory models of research utilization.

Model	Characteristics	Shortcomings
Model 1 **Knowledge push explanation **[14,15,18-20]	• Assumes linear sequence from supply of research to utilization by decision makers • Assumes that high quality research will automatically lead to higher uptake and use by decision makers • Content attributes of the research influence its use by decision makers. For example: notability, complexity, validity and reliability • Type of research influences its use by decision makers. For example: theoretical/applied, quantitative/qualitative, research domains and disciplines	• No acknowledgment of the incremental nature of policymaking, • Quality is a necessary, but not sufficient, condition for user's attention. • It is not always clear who takes responsibility for transfer, • There is a process of transforming academic knowledge into useable knowledge

Model 2 **Dissemination explanation **[14,15]	• Assumes linear sequence from supply of research to utilization by decision makers • Recognizes the fact that knowledge transfer is not automatic. • Suggests that an extra step should be added to research activities by developing dissemination models. It suggests developing a strategy to disseminate research results. • Type of research output (results) explains research utilization • Dissemination efforts explain research utilization	• Assumes "unidirectional" dissemination from producers to users. • Includes neither the involvement of potential users in the selection of transferable information nor involvement in the production of research data.

Model 3 **Demand pull explanation **[14,15,21]	• Assumes a linear sequence from supply of research to utilization by decision makers • The initiative is shift to the policy makers. As such, this explanation asserts that as policy makers identify problems and define the needs, they ask researchers to conduct studies that will generate alternatives or solutions. • Knowledge utilization is explained by the needs of users.	• No acknowledgement of the incremental nature of policymaking. • Does not consider the fact that the results of necessary research can be pushed aside because they do not stroke with personal or organizational interests • Omits the interaction between producers and users of research findings.

Model 4 **Organizational interests explanation **[14,15,22,23]	• Assumes a linear sequence from supply of research to utilization by decision makers • Variant of Demand Pull Explanation • Stresses that personal and organizational interests are important impeding factor for research utilization. • Important factors are organizational structures, types of policy domains, needs of organizations and positions of actors. • Within this explanation, the use of knowledge increases "as users consider research pertinent, as research coincides with their needs, as users' attitudes give credibility to research and when results reach users at the right time".	• No acknowledgement of incremental nature of policymaking • Places too much emphasis on the interest of users and neglects the fact that users do not merely act as rational consumers, looking for their own profit. Users have also irrational preferences, belief systems and values

Model 5 **Two communities explanation **[14,15,17,22,24-30]	• Assumes a cultural gap between researchers and users, which is visible in different communities, different language and different methods of communication • Adaptation of research products by users reduces the cultural gap utilization; therefore researchers should invest in more readable and appealing reports, make more specific recommendations and focus on factors amenable to interventions by users • Acquisition efforts by research users reduce the cultural gap. This means that users are making an effort to influence the research agenda by discussing the subject and scope of research projects with researchers and discuss results.	• No assumption about the process, either linear or incremental. • Emphasizes the cultural gap and pays no attention to factors mentioned above • No attention for the influence of the construction of the policy network, advocacy coalitions an institutional constellations

Model 6 **Interaction explanation **[14,15,30-35]	• Offshoot of the Two Communities Explanation and is analogous to the elected affinities model. • The process is a set of interactions between researchers and users, rather than a linear move from research to decisions • This explanation suggests that research utilization is brought about by various interactions between the researchers and the policy makers. Interaction does not start with the needs of researchers or needs of policymakers. • It is assumed that the more sustained and intense interaction between researchers and users, the more likely utilization will occur. • Important factors are the so-called linkage mechanisms and dissemination efforts	

There are two main rational explanation models of research utilization: knowledge push [14,15,18-20] (model 1) and demand pull [14,15,21] (model 3). Both assume a linear sequence from supply of research to utilization by policy makers. This assumption is a weak point of the explanations because of the incremental nature of the policy development process. The initiative for use lies either with producers (researchers) or with users (policy makers).

Two other explanations are complementary to the aforementioned explanations: the dissemination explanation [14,15] (model 2) elaborates on the science push explanation, as the organizational interests' explanation [14,15,22,23] (model 4) elaborates on the demand pull explanation. Caplan's 'two communities' explanation [14,15,17,22,24-30] (model 5) takes a different approach. It emphasizes the cultural gap between researchers and policymakers, which Jansen refers to as "niches" [1]. Caplan argues that it is necessary to frame research outcomes in such a way that these fit in the niche of policymakers. Furthermore, Caplan's explanation model suggests that it is also necessary for policymakers to be involved with research agendas and design [24]. However, there is also a critique of this explanation. Lin and Gibson argue that "the two communities alone is an inadequate basis for attempts to change the way research and policy relate to each other" [17]. They question whether the model captures important determinants like the rejection or acceptance of research by advocacy coalitions during policy development based on their core values and beliefs, the influence of institutional structures within policy networks and the perspective that researchers already make part of the policy makers domain and that the so called 'gap' does not exists.

The final explanation model focuses on the interaction between researchers and policymakers [14,15,30-35] (model 6). Interaction can be defined as the reciprocal actions of two or more people who work together, negotiate on opinions, values and norms and find consensus. The explanation assumes that the presence of interaction and how interaction takes place, explains the way research is utilized during policy development.

### Identifying specific barriers between policymakers and researchers

To elaborate on these six types of explanatory models, table 2 provides an overview of the seventeen barriers found in the literature and the inventory of RHSs. In the third column of table 2, critical key factors of influence derived from the barriers are shown. Based on the findings we made a distinction between barriers at the *process level *and at the *individual level*. The process level refers to barriers related to the different steps and phases in the research process. The individual level refers to barriers related to characteristics of (policy) receivers of research information.

**Table 2:** Overview of barriers in research utilization.

Specific barriers	Lit ref	Identified critical key factors of influence	Problem level	Problem domain
1. No awareness of researchers about the policy process	[12,21,27,36] and mentioned in inventory	Creating insight in working processes	Process	Expectations (Preparation phase of research)

2. Finding researchable questions	[7,12,27,29,30,33,37,38] and mentioned in inventory	Negotiate research questions, make an inventory on the need of information	Process	Expectations (Preparation phase of research)

3. Answers about a specific item	[12,30,39,40] and mentioned in inventory	Discuss limitations of study design and timelines	Process	Expectations (Preparation phase of research)

4. Limited results by choice of study design, mostly cross-sectional studies, no causes and solutions	[12,27,39,40] and mentioned in inventory	Discuss limitations of study design and timelines	Process	Expectations (Preparation phase of research)

5. Degree of uncertainty	[12,21,27,39]	Discuss limitations of study design and timelines	Process	Expectations (Preparation phase of research)

6. Actuality	[12,21,27,39] and mentioned in inventory	Discuss limitations of study design and timelines	Process	Expectations (Preparation phase of research)

7. Timing	[7,12,21,25,27,30,33,38,39,41-43] and mentioned in inventory	Which research information is given at what time	Process	Expectations (Preparation phase of research)

8. Language	[12,18,22,27,33,38,39,44,45] and mentioned in inventory	For which target group is the information intended; what jargon is used How convincing is the research message How is the package What is the relation with other policy domains	Process	Transfer (Publication phase of research)

9. Conflicting knowledge by other Researchers	[39,40,42,46]	Collecting other research information	Process	Transfer (Publication phase of research)

10. Media	[12,43,47]	Communicating with media	Process	Transfer (Publication phase of research)

11. Perceived robustness of evidence	[15,22,25,41,45,46,48-50]	How do stakeholders perceive the quality of the research	Individual	Acceptance

12. Perceived credibility of source: researchers or other stakeholders	[25,28,29,38,41,43,51,52] and mentioned in inventory	Who is bringing the message	Individual	Acceptance

13. "Fit" with personal knowledge, values or belief systems, preferences and traditions	[25,41,43,46,50,51] and mentioned in inventory		Individual	Acceptance

14. Consider whether or not a problem is important enough to deal with, relevance	[21,25,32,41,43,46,50,51]		Individual level	Interpretation

15. Consider connection with own personal or institutional interests	[21,25,32,41,43,46,50,51]		Individual level	Interpretation

16. Consider whose responsibility it is to take action	[21,25,32,43,46,50,51]		Individual level	Interpretation

17. Consider which solutions are at hand	[21,25,32,43,46,50,51]		Individual level	Interpretation

The process related barriers were classified in two domains: the *expectation *domain and the *transfer *domain. In the *expectation *domain [12,21,25,27,29,30,33,36-42] we classified barriers that can be acted upon during the preparation phase of research. This domain addresses the issue of awareness among researchers and policymakers of each other's 'niches'. The second domain of *transfer *[12,18,22,27,33,38-40,42-47] addresses how research is communicated and the involvement of the media. This domain refers to the publication phase of the research cycle. Also the barriers at the individual level were classified in two domains: the *acceptance *domain and the *interpretation *domain. Barriers classified under *acceptance *[15,22,25,28,29,41,43,45,46,48-52] refer to the degree to which a person believes the research outcome to be true; not about the scientific validity or credibility, but the perception of these by researchers and policymakers. Barriers classified under *interpretation *[21,25,32,41,43,46,50,51] deal with the value people give to research outcomes, in this case local health problems. In other words "is the problem important enough to act?" The value of research outcomes depends on personal experiences and interests, organizational interests and possibilities of (policy) solutions.

### Concepts of research utilization or research impact

The extent of research utilization or research impact can be assessed in different areas, like in the scientific area, policy area, health services and organizational area and societal area [53].

Within the policy area, there are two main concepts found in the literature regarding research utilization and impact. The characteristics of the concepts are stated in table 3. The first concept is derived from Amara et. al. [22] and is partly based on the earlier work of Weiss [39]. They distinguish three types of research utilization models: instrumental, conceptual and symbolic. Other authors accepted these three types of use and have even delineated subtypes [2,6,32,53]. The second concept stems from Knott and Wildavsky in 1980 [54] and is called "the ladder of research utilization'. As shown in table 3, it distinguishes seven stages and suggests a normative degree of research utilization - the higher the step, the better [13,31]. If we compare the two concepts, Amara et.al. on the one hand and Knott and Wildavsky on the other, it seems that the "instrumental use" of Amara et.al. overlaps with the highest stages of implementation and impact from Knott and Wildavsky. The "conceptual use" overlaps with "reference" stage of the research utilization ladder. The last type of use defined by Amara et.al., "symbolic use", does not seem to fit directly into the research utilization ladder.

**Table 3:** Two main concepts of research use.

Concept of research utilization		Description
**Types of research utilization **[6,22,32,39,40]	Instrumental	When research is acted upon in specific and direct ways, i.e. to solve a problem at hand
	
	Conceptual	Contributing to improved understanding of the subject matter, related problems, more general and indirect form of enlightenment
	
	Symbolic	Justify a position or course of action for reasons that have nothing to do with the research findings (political use) or use the fact that research is being done to justify inaction on other fronts (tactical use)

**Ladder of research utilization **[13,31,54]	1. Reception	Research results are received by actors
	
	2. Cognition	Research results are read and understood
	
	3. Reference	Research results change a way of thinking by actors
	
	4. Effort	Efforts are made to get the research results into policy even when this was not successful
	
	5. Adoption	Research results has direct influence not only on the policy process but on the context of the policy
	
	6. Implementation	Research results not only has been used for policy formulation but also translated into practice
	
	7. Impact	This refers to successful implemented policy initiated by research results.

## Towards a conceptual analytical framework

The purpose of this article is to identify a useful analytical framework for research utilization in the Dutch setting of local health policy development, and to use it for further empirical studies in this field.

In the literature we see the interaction model is internationally regarded as the main explanatory model [13,30,32,53]. It acknowledges the interactive and incremental nature of policy development, which takes place in a context that includes diversity within the groups of researchers and policymakers regardless how they are organized. The elected affinity theory of Short is related to the interaction explanation. This theory assumes that the extent of contact and timing of interaction between researchers and policymakers and the fit with personal values and beliefs will improve a positive reception from the policy audience [35]. Also the linking and exchange model developed by Lomas [19] focuses on mutual exchange and the joint creation of knowledge between policy makers and researchers. Here we see a link between interaction and the overview of barriers we presented. The theory of Short and the model of Lomas presume that interaction can avoid barriers and in this way improve research utilization. So assuming a network of policy stakeholders, different barriers can occur with different stakeholders. Then it becomes interesting to study when and with whom interaction takes place, in what way and with what result.

In addition, de Leeuw et al provide useful theoretical models in which they describe the different ways the "barriers" between research and policy can be overcome [55]. They distinguish between seven models which can be ordered in three groups. First of all there is a theoretical model regarding changing the rules and games within the structure of the research and policy networks called "the institutional re-design" model. Secondly there are four theoretical models about the ways interaction takes place and the nature of the evidence: the "Blurring the boundaries" model which is about the reciprocal participation of researchers in the policy process and of policymakers in the research process; the "Utilitarian Evidence" model in which research outcomes are articulated in a way that reflects current political agendas; the "Conduit" model about the role intermediaries play between research and policy; and the "Alternative evidence" model which is about the importance of more supporting evidence so that the research outcomes can no be longer ignored even if the issues is not on the policy agenda. Thirdly, two theoretical models about the ways of communication are distinguished: the "Research narratives" model in which research outcomes are made personal and the "Resonance" model where interaction is intended to connect with underlying belief systems of policymakers [55].

The interaction models above are related to domains in our conceptual framework. For example "Utilitarian evidence" and "Research narratives" are related to the *transfer *domain, while the "Resonance" model relates to the *acceptance *domain.

In the background section we explained the dynamic and complexity of context of Dutch local health policy. Researchers and policymakers are influenced by the culture of the institutions they work in. Researchers act and make decisions in the research process in keeping with the norms of a specific research institute. This implies that researchers working in the RHS setting are influenced by their fellow researchers and other local public health professionals. Policymakers on the other hand must consider multiple actors in the policy process. These actors can, for example, be civil servants or local administrators, members of the city council (politicians), professionals of public services from related policy domains or representatives from interest groups.

In the conceptual framework, not only interactions between a specific researcher and a specific policymaker must be considered, but also interaction between other actors within and between the research and policy process. Therefore we propose for our conceptual framework a network approach, in which we "extend" the interaction model. We not only focus on the one-to-one relationships between an individual researcher and policymaker but include interactions between several actors participating in the research and policy process.

In policy and administration sciences there are different perspectives on how to study the policy process. The network perspective provides theoretical concepts and normative starting points for analyzing and assessing complex processes of problem solving in network settings and the roles that perceptions, interactions and institutions play in this [56]. Policy networks have a number of characteristics [2,57-59]:

• Variety of actors in terms of size, interests, power and perception of problems;

• Reservations on the part of individual actors, the willingness to cooperate and their strive for autonomy;

• Mutual dependencies between the actors on each other's resources and decisions;

• Fragmented problem solving ability where actors also depend on each other's resources and;

• Coordination by bargaining where decisions are a result of consultation and bargaining processes.

Stone [44] suggests that research can play a key role in the policy process when researchers are network participants. Also Nutley agrees that concepts of policy networks provide a useful framework to study the context of policymaking and research utilization [60]. They even say that the looser the policy network, the more divergent are the views represented and the wider the range of different types of research that are likely to be used by those advocating different policy lines. However there is also substantial critique on the network theory [61]. It is argued that it is only a way of describing the policy process, but it explains little about how the network actually influences the policy process itself.

**Figure 2:**
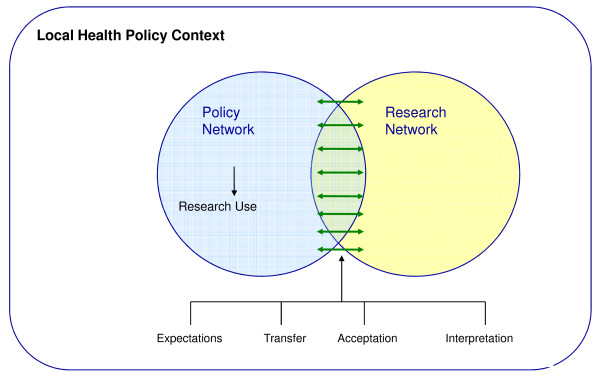
**Analytical framework for analyzing use of epidemiological research for local health policy development**.

To conclude, in our conceptual framework the network approach offers a frame to describe the policy process and respectively research utilization. It will show us how the arena is shaped and whether this influences the presence or absence of interaction between actors and existing barriers. Subsequently the elements of interaction and barriers have to provide an actual explanation of research utilization. We think it is of interest not only to take a network perspective on the policy process but also on the research process. In figure 2, the proposed analytical framework is presented. We visualize the research and the policy networks both as circles. In the research network actors are researchers or health professionals, working together on a research project, discussing questions, design, analytic strategies or papers. In the policy network actors discuss and negotiate on the importance of public health problems and the possible solutions at hand. Here we also find different actors, some in power over others, some with financial resources and others with specific knowledge and expertise. Actors may exchange information or choose not to do so. There is a possibility of overlap between the networks. This happens when policymakers get involved in the research process, for example when formulating research questions, or researchers are participating and communicating their results in the policy process. Notably this type of interaction relates to the model, "Blurring the boundaries" of de Leeuw et al [55]. How these processes of research and policy are organized, the constellation of the research and policy networks and the way interactions between actors both within and between these networks appear, are empirical questions.

To understand research utilization it is important to study the presence of the aforementioned factors in the policy context, barriers in communication, the constellation of the network and the behaviour of actors and the interaction between them. From this point of view it seems evident to differentiate between research utilization of an individual actor and utilization within the policymaking process [4]. The first would mean that individual actors within the policy network use research information in the policy-making process. The three types of research utilization as proposed by Amara et.al. [22] could be a good indicator for this. Utilization on the process level would mean the impact of the research information within the policy process. An adapted and more elaborated ladder of research utilization [54] could be the base for a feasible instrument for this purpose. Impact can be measured whether information is disseminated, read and discussed by policy actors up to successful influence of it on policy itself.

## Conclusions and Future work

This article shows a substantial number of critical key factors that contribute to or impede the use of epidemiological research in local health policy making in the Dutch political context.

The developed framework has added value on existing analytical frameworks and models like Landry [14] and Hanney [15] because it emphasizes more on the 'logic' of the context and the existing networks within this specific public health policy domain. By 'logic' we mean the aims, duties and responsibilities of actors from participating organizations and relations between them in the Dutch context of local health policy. The choice for this approach is internationally mentioned before and recommended [62]. The framework gives the opportunity to take the possible effect of this logic on the use and impact of research for local health policy development into account. Recent Dutch studies showed that, on the national policy level, the different interactions between researchers and policymakers during the research and policy processes provides useful insights [9,63].

As stated at the beginning of this article the proposed conceptual framework is to be used in empirical studies about how epidemiological research progresses within the policymaking process. The primary research question in these studies is whether or not interactions will contribute to the use epidemiological research in local health policy development. To obtain more insight into this, we will first conduct in-depth case-studies in three municipalities and their RHS, using social network analyses. Secondly, we will make a national description of the impact of epidemiological research on local health policy making within Dutch municipalities and the interaction between them and their RHS.

Further operationalisation of the concepts mentioned in the framework is necessary. Different contextual and key factors have to be transformed into relevant questions for actors about their position in the networks, their relations, their involvement in research, their attitude towards it and their perception and judgment on the way research was transferred. On the one hand we will study existing barriers described in the conceptual framework, on the other hand we intend to elaborate on the theoretical models of de Leeuw et al, and how the barriers are overcome in the empirical situation [55,64]. Also the way impact and use of research is measured needs further elaboration in questions. Therefore we intend to adapt and translate earlier used questionnaires by Amara [22], Landry [31], and Kothari [26].

We expect the results of these studies will contribute to a better understanding of the use or impact of local epidemiological research in local health policy development and the role of researchers within this development.

## Competing interests

The authors declare that they have no competing interests.

## Authors' contributions

JDG has drafted the manuscript and designed the study, acquisited the data and analyzed and interpreted the data. KP, TVDG and HVO have revised the manuscript critically for important intellectual content. All authors have given final approval of the version to be published.
